# A New Apparatus for Recording Evoked Responses to Painful and Non-painful Sensory Stimulation in Freely Moving Mice

**DOI:** 10.3389/fnins.2021.613801

**Published:** 2021-02-12

**Authors:** Jiaojiao Zhang, Lee Embray, Yevgenij Yanovsky, Jurij Brankačk, Andreas Draguhn

**Affiliations:** Institute of Physiology and Pathophysiology, Medical Faculty Heidelberg, University of Heidelberg, Heidelberg, Germany

**Keywords:** freely moving mice, evoked potentials, behavioral test, laser, pain, tactile, somatosensory

## Abstract

Experiments on pain processing in animals face several methodological challenges including the reproducible application of painful stimuli. Ideally, behavioral and physiological correlates of pain should be assessed in freely behaving mice, avoiding stress, fear or behavioral restriction as confounding factors. Moreover, the time of pain-evoked brain activity should be precisely related to the time of stimulation, such that pain-specific neuronal activity can be unambiguously identified. This can be achieved with laser-evoked heat stimuli which are also well established for human pain research. However, laser-evoked neuronal potentials are rarely investigated in awake unrestrained rodents, partially due to the practical difficulties in precisely and reliably targeting and triggering stimulation. In order to facilitate such studies we have developed a versatile stimulation and recording system for freely moving mice. The custom-made apparatus can provide both laser- and mechanical stimuli with simultaneous recording of evoked potentials and behavioral responses. Evoked potentials can be recorded from superficial and deep brain areas showing graded pain responses which correlate with pain-specific behavioral reactions. Non-painful mechanical stimuli can be applied as a control, yielding clearly different electrophysiological and behavioral responses. The apparatus is suited for simultaneous acquisition of precisely timed electrophysiological and behavioral evoked responses in freely moving mice. Besides its application in pain research it may be also useful in other fields of sensory physiology.

## Introduction

Defined by the International Association for the Study of Pain (IASP), pain is an unpleasant sensory and emotional experience associated with, or resembling that associated with, actual or potential tissue damage (International Association for the Study of Pain (IASP), 2020). Despite its subjective nature, pain has clear physiological correlates in both the central and peripheral nervous system. In the mammalian brain, pain goes along with the simultaneous activation of several cortical and subcortical areas which, together, form a “pain network” or “pain matrix” (Apkarian et al., 2005; Gross et al., 2007; Dowman et al., 2008; Zhang et al., 2012). This does, in principle, open the possibility to define electrophysiological markers of pain states, i.e., specific patterns of activity in pain-processing neuronal networks. Until now, however, such a neurophysiological signature of pain is missing, despite its enormous expected clinical benefits. It appears that the pain-specific signal content vanishes in the superimposed basal neuronal activity which, in EEG recordings, takes the form of state-dependent widespread network oscillations. It might therefore be promising to begin with a clearly delineated signal by measuring temporally and spatially restricted responses to acute painful stimuli. Such evoked potentials provide reproducible, modality-specific patterns which can be averaged from multiple trials to yield sufficient signal-to-noise separation.

In human studies, brief infrared laser pulses are widely used as nociceptive stimuli, and laser-evoked potentials are regularly recorded in pain studies using methods like EEG or ECoG (Bromm and Treede, 1984; Cruccu and Garcia-Larrea, 2004; Ohara et al., 2004; Rosenberger et al., 2020). Several experiments on animals have also used laser-evoked potentials, including ECoG recordings from unrestraint rats (Ljungquist et al., 2016; Peng et al., 2018). However, most present approaches suffer from technical limitations including the lack of high-resolution recordings from subcortical networks, behavioral restriction of the experimental animal, or missing possibilities to apply stimuli of other modalities. An important condition for yielding reproducible evoked potentials is the precise temporal alignment of responses. This is particularly difficult when using behavioral responses to heat stimuli as a temporal marker, such as in the hot plate test, the Hargreaves test and the thermal probe test (Hargreaves et al., 1988; Deuis et al., 2017). While it is possible to align withdrawal reflexes with the electrographic responses (Zandieh et al., 2003; Hill et al., 2009; Ljungquist et al., 2016; Inada et al., 2020), latencies are often on the scale of seconds while evoked potentials follow neuronal activation on a much faster time scale. Therefore, the alignment and averaging of evoked potentials on the basis of behavioral responses can be noisy. One approach is to use devices with noxious stimulation inducing very prompt behavioral reflexes (Bromm and Treede, 1984). Here we describe another approach, aligning electrographic responses with precisely measurable cue times and using behavioral data only for the assessment of pain.

We therefore constructed an apparatus combining laser-induced short-latency behavioral responses with measurements of evoked potentials from different brain regions. In addition, the method allows application of other kinds of stimuli, e.g., tactile stimulation, for comparison. Importantly, the animal is not restricted, avoiding additional stress and allowing natural behaviors such as paw withdrawal, guarding, freezing, or flinching (Luger et al., 2002). Our results show that graded pain-related evoked potentials can be reliably recorded from freely moving mice. Responses to laser-evoked painful stimuli are different from non-painful tactile stimuli, both behaviorally and electrophysiologically.

## Materials and Equipment

### Custom Made Apparatus for Sensory Testing

**FIGURE 1:**
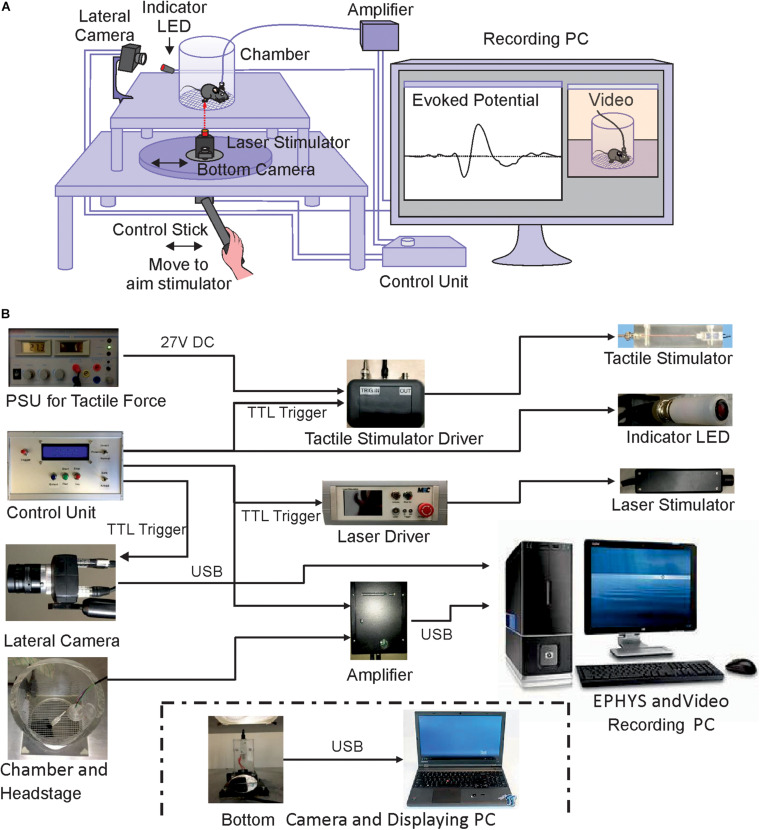
Schematic illustration of the experimental setup. **(A)** Structure and connections of the custom-built apparatus. **(B)** Assembly of components. Power supplies, lateral camera and control units are shown on the left, interfaces and recording amplifier in the middle part and stimulation devices and computer for data storage on the right. The bottom camera and displaying laptop for positioning the stimulator underneath the mouse paw are shown separately below the other components.

**FIGURE 2:**
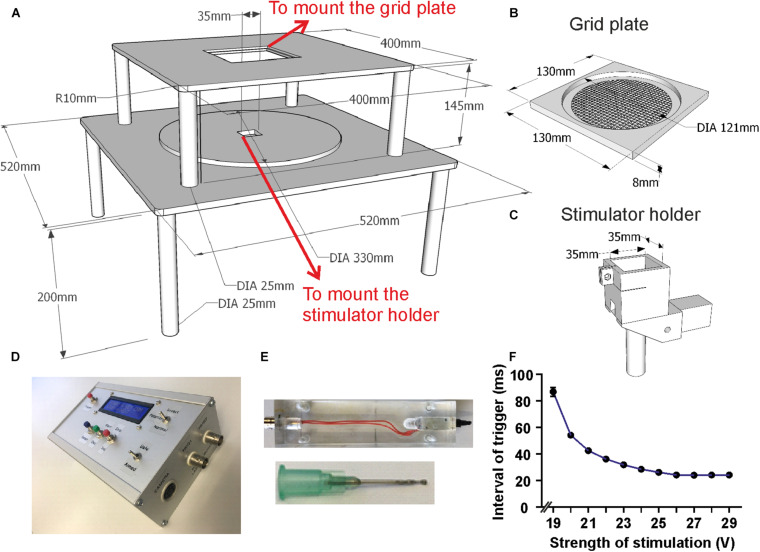
Setup dimensions and details of components. **(A)** Schematic drawing of the main scaffold with all dimensions marked. **(B)** Schematic drawing of the grid plate which can be embedded into the upper platform. **(C)** Design of the holder for laser/tactile stimulators which fits into the hole in the lower layer of the scaffold. **(D)** Photo of the custom-made control unit. **(E)** Photos of the custom made tactile stimulator and its blunt needle. **(F)** Delay time between trigger and mechanical response to tactile stimulator. The interval decreases as the stimulation strength increases and stabilizes for stimulation voltages from ∼27 V. Mean with SEM.

The device was custom built from stainless steel, consisting of two stapled square base plates of 400 and 520 mm side length, respectively (Figures 1A, 2A). Both platforms were supported by legs and contained square holes in the middle (side length 130 mm for the grid plate holder and 35 mm for the stimulator holder). A freely sliding round plate was positioned on the bottom plate to suspend a stimulator and a camera (Figures 1A, 2A). The top plate was fixed at 345 mm above the base plate, securing the required height for operating the laser stimulator (MRC Systems GmbH, Germany). Stimulators (tactile and laser) were required to be effortlessly moved to the correct position underneath the hind paws of the mouse. This was achieved by coating the base plate with self-adhesive PTFE Glass Fabric (High-Tech-Flon, Konstanz, Germany) reducing friction.

We used an infrared laser stimulator (MRC Systems GmbH, Germany) of 1470 nm and with pulse durations from 1 – 100 ms (pulse energy 1.2 – 306 mJ) (Figure 1B). A pilot laser of 650 nm (red light, visible to naked eye) indicted the position of the laser beam. The light source was mounted on the sliding plate, and the beam (initial diameter 4 mm) was focused by an adjustable lens to 50 μm diameter (∼area 2000 μm^2^) in the focal plane which was at 30 mm above the laser. Using illumination times of 3 – 10 ms the applied energy per trial was 1.5 – 6 mJ/mm^2^, comparable with published data on rodents (Mitchell et al., 2014) and humans (Rosenberger et al., 2020). The exact vertical position of the laser stimulator was adjustable by a screw clamping it into the holder/control stick assembly (Figures 1A, 2C). The laser source was easily removable from the holder and could be replaced with other stimulators, for example the custom built tactile stimulator (see details below) which had exactly the same mounting dimensions as the laser (Figures 2C,E). The horizontal position of the stimulator could be freely determined by manually moving the connected control stick (Figure 1A). A vertically directed camera was mounted to the stimulator holder allowing a direct view from underneath to align position with the hind paw of the animal (Figures 1A,B).

A circular grid plate (100 mm in diameter) was produced by a 3D printer (Formlabs Form2, United States) and inserted in the hole in the top plate to create a floor for the animal that allows laser/tactile stimulation to be conducted without obstruction (Figures 2A,B). A transparent plexiglass cylinder (120 mm in diameter) was then placed vertically on top of the circular grid, bordering the experimental arena of the mouse (Figure 1A). The space inside the chamber was large enough to allow unrestricted behavior of the animal, yet small enough to keep the paws within convenient reach for the laser or tactile stimulator. The top of the chamber was left open for the preamplifier (headstage) to be connected to the amplifier board (Intan Technologies, United States) above the chamber. A HD-camera (iDS, Germany) was mounted close to the chamber to document the behavior of the mouse (Figures 1A,B). Video images (14 fps, display resolution 1,280 × 1,024) were collected with μEye Cockpit software (iDS, Germany) on a standard computer (Figure 1A). We note that this speed is not sufficient for detailed monitoring of motor behavior, including a precise measurement of response latencies. An in-depth analysis of pain-induced reflexes requires high-speed video recording (e.g., Mitchell et al., 2014) which can be easily added to the apparatus but is beyond the scope of the present experiments. The camera also recorded a red indicator-LED light on top of the mouse platform indicating when the laser or the mechanical stimulation had been triggered. This signal facilitates synchronization of stimulation and recording, measuring paw withdrawal latency and aligning evoked potentials. The LED light was directed toward the lateral camera and was not visible to the animal to be sure not to trigger any behavioral reaction, confirming that the LED and the laser light themselves provided no relevant cues. This was also confirmed by the different responses to laser- and mechanical stimuli which both activated the LED in the same manner. Evoked potentials and video signals were presented and saved on a connected computer for offline analysis (Figures 1A,B).

### Control Unit

**FIGURE 3:**
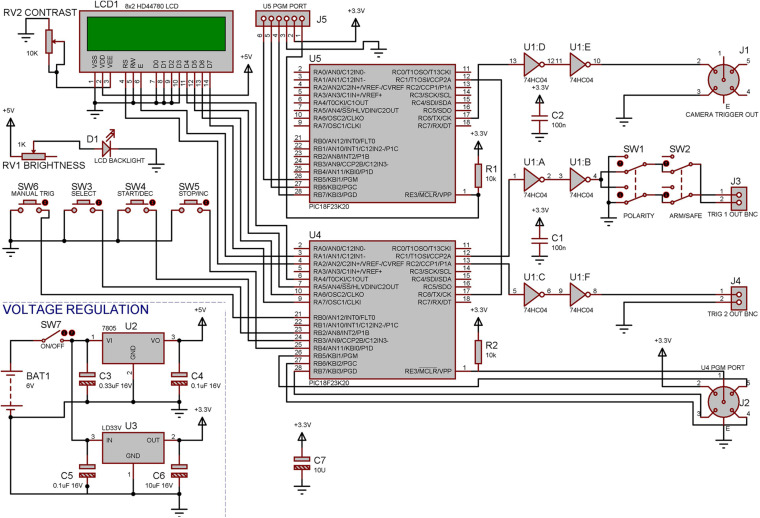
Electronic design of the control unit. The controller was built using two PIC18F23K20 Microcontrollers, one of which (U4) was used to perform all functions that are not time critical such as running the Menus, LCD display, and the two Trigger outputs, the second microcontroller (U5) had the sole responsibility of producing an accurate frame trigger for the camera allowing this to be synced up later a with real time clock. Both of the microcontrollers can be reprogrammed in circuit using provided connectors allowing easy modifications to the program at any time.

A separate control unit (Figures 2D, 3) was designed to handle the following functions:

i. Triggering of the laser/tactile stimulator either manually or automatically;
ii. Triggering the red LED indicator (in view of the lateral camera);
iii. Controlling recording intervals of the lateral camera as required by the experiment;
iv. Initiate trials with random timing within a pre-set minimum and maximum time interval.

The design of the control unit (Figure 3) is based on two PIC18F23K20 microprocessors (MicroChip, United States) which were programed in C using MPLAB X IDE (MicroChip, United States). One microprocessor performs all menu options and standard TTL outputs and the second is dedicated to accurately triggering the correct frame rate for the camera. The device is battery powered to reduce any extra electrical noise from an external power supply. All parameters can be set via a simple two-line LCD display and three menu buttons. The two main trigger outputs can be adjusted to durations between 1 to 250 ms. Trigger one was set to 1 ms and is used to trigger the laser unit (laser-on duration is set on the laser driver itself). Trigger two was set to 100 ms and is used to drive the red LED in view of the lateral camera (100 ms is sufficient time to produce a reliable video signal when the laser is triggered), and this trigger was also fed into the electrophysiological recording system for synchronization with the LED-on period in the video. As for the automatic trigger function, the pre- and post-record times (pre-record maximum of 16 s, post-record maximum of 240 s) were set to 3 and 30 s accordingly meaning the video would start recording 3 s before the stimulation and continue recording 30 s after the stimulation.

A Safe/Armed switch was required to eliminate accidental triggering of the system (accidental manual trigger button pressing), avoiding possible dangers for mouse and operator as well as recording unwanted data. A polarity switch was added in case other equipment was to be utilized that may require an inverted trigger pulse rather than a positive trigger pulse.

The control unit can be triggered manually or drive the experiment autonomously, with the exception of targeting the stimulator to the mouse paw. The experiments shown below have been performed with manually triggered cues.

All electrophysiological and video data was recorded on a PC for later offline analysis (Figure 1).

### Tactile Stimulator

**FIGURE 4:**
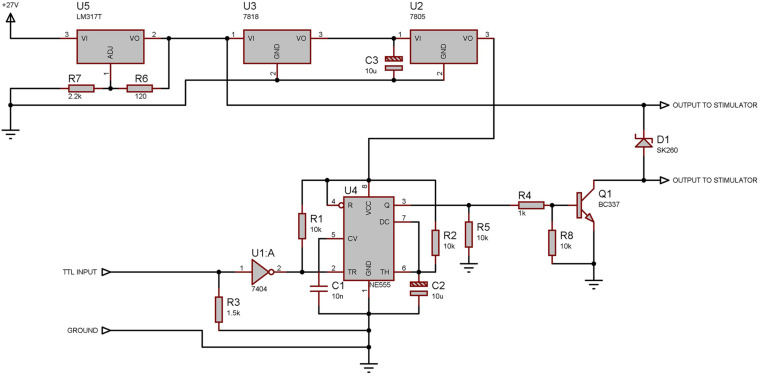
Electronic design of the tactile stimulator. The 27 V signal is reduced to 5 V to power the NE555 chip which is responsible for the duration of the pulse sent to the actuator. U5 regulates the output voltage to 24 V when the input voltage is equal to, or more than 27 V: Using an input voltage of less than 27 V reduces the force applied to the actuator and increases the actuation time.

A tactile stimulator was constructed in-house to be interchangeable with the laser stimulator. For this reason it has exactly the same outer dimensions as the laser module (Figures 1B, 2E). The stimulator is based on a solenoid whose shaft was equipped with a conical tip. At this position, any standard hyperdermic needle is easily mounted, allowing to use a wide range of different thicknesses and lengths as well as sharp or blunt tips. In our recordings, a blunt 0.8 mm × 22 mm needle (Sterican, B. Braun, Germany) was modified with a 1mm silver solder ball attached to the tip. The circuit of the tactile stimulator is shown in Figure 4.

The 12 V solenoid (EBE Group TDS-04C) was sandwiched between two machined Plexiglas halves creating the body of the stimulator while protecting the solenoid and making cleaning easier. Outer dimensions of the two Plexiglas halves together match those of the laser stimulator allowing for simple exchange. The connections to the solenoid are terminated with a BNC connecter at the end of the body, opposite to the needle. A driver unit was constructed to be triggered by the TTL signal from the controller and drive the solenoid at a selected voltage provided by any external adjustable laboratory power supply capable of supplying up to 30 V. Voltage was supplied to the solenoid for duration of 100 ms.

While the solenoid was rated at 12 V, it could be operated at much higher voltages (Max 30 V, Typically 27 V) for our very short stimulation times, given that the long periods of time between use allow for cooling of the solenoid winding.

Tests were performed to measure the time taken between the TTL signal input and the physical stimulation of the paw. This was carried out using a piezo sensor positioned in place of the paw connected to the first channel of an oscilloscope (Textronix TDS2024 United States), while the TTL input signal was connected to the second oscilloscope channel. We then measured the duration between the two signals for various voltage settings across the solenoid (Figure 2F).

A voltage of 27 V created reproducible and sufficiently strong movements of the needle during minimal operating time. At this voltage, the time between TTL pulse and physical stimulation was measured at a constant 24 ms. This delay was subtracted from the measured behavioral response latencies and was used to synchronize all recorded video and electrophysiological data for tactile stimulation (Figure 2F). Using higher voltages had no further effect.

## Methods

### Ethics Statement

The experiments were performed under the guidelines of the European Science Foundation (2001), and were approved by the Governmental Supervisory Panel on Animal Experiments of Baden Württemberg, Karlsruhe (35-9185.81/G-115/14). All efforts were made to use minimum number of animals for this study and to minimize the animal suffering. All experiments involving laser light were done in a special room with a warning light above the door. Experimentalists wore protection googles during the experiments.

### Animal Housing

Nineteen male C57BL/6N mice were purchased from Charles River Laboratories (Sulzfeld, Germany) at an age of 10–12 weeks. Before surgery, the animals were housed in groups of two in a ventilated Scantainer (Scanbur BK, Denmark) on a normal 12/12-h light/dark cycle, with free water and food supply. After surgery each mouse was housed separately to prevent electrode damage, and changed to an inverted 12/12-h light/dark cycle. After completion of all experiments the mice were sacrificed by an overdose of pentobarbital (i.p.) during brain perfusion (see below).

### Electrode Preparation

Electrodes for intracerebral field potential recordings were constructed from two insulated tungsten wires (diameter: 50 μm, California Fine Wire, United States) which were glued together and cut to approximately 2 cm length. Insulation was scratched off from one end which was soldered to a copper pin (Farnell, Germany) and connected with the preamplifier (headstage).

Surface electrodes were prepared from stainless steel watch screws (diameter: 1 mm, length: 3 mm, DIN84, Wegertsender, Germany). Insulation of copper wire (diameter: 0.22 mm, length: 20 mm, Conrad, Germany) was removed from both ends. One end was soldered to a stainless steel watch screw (1 mm diameter) and the other to a gold-plated pin (Farnell, Germany) for connection with the headstage. The screw was fixed in the skull during surgery (see below) such that it gently touched the surface of the brain.

### Surgery for Electrode Implantation

Animals were anesthetized with 4% isoflurane (Baxter, Germany) together with medical oxygen (float rate: 1 L/min). After ceasing of the righting reflex, buprenorphine hydrochloride (0.1 mg/kg bodyweight, Indivior United Kingdom Limited, United Kingdom) was injected subcutaneously from a stock solution (0.324 mg/ml in 18.5 ml sterile 0.9% saline). This injection was repeated at 8 h after surgery to prevent postoperative pain. During surgery, anesthetized animals were mounted into a stereotaxic frame (David Kopf Instruments, United Kingdom) under ongoing anesthesia with 1.0 – 2.5% isoflurane. Body temperature was kept at 37 – 38°C by a heating pad (ATC-2000, World Precision Instruments, United Kingdom) and spontaneous breathing rate was checked every 10 min. After shaving the hair on the head the skull was exposed with a scalpel and the skin fixed with surgical thread (Vicryl V734E 4-0, Ethicon, Germany). Subsequently, holes were drilled at defined locations according to the coordinates from Paxinos and Franklin (2001) using bregma as reference. Up to 13 electrodes (including ground and reference) were implanted in individual mice. Target regions included contralateral and ipsilateral primary somatosensory cortex (S1c and S1i), anterior cingulate cortex (ACCc and ACCi), ventral posterolateral thalamic nucleus (VPLc and VPLi), posterior insula (Insc and Insi), central nucleus of the amygdala (AMYGi), olfactory bulb (OBi), and parietal cortex (PACi). Stereotactic coordinates of the targeted networks are shown in Table 1. Grounding (GND) and reference electrodes (REF) were screwed to the surface of the cerebellum (Figure 2A). Electrodes were fixed with composite dental fillers (Filtek Supreme XTE, 3M, United States) and with dental cement (Paladur, Heraeus GmbH, Germany). All contact pins were inserted into a dummy female connector for assembly and protection. This procedure ensured stable electrode position and recording conditions throughout the experiments. Correct electrode positions were controlled by histological staining after the experiment (see section “Histology”). After surgery, the animals were placed into their cage and the environment was maintained at 28°C until the animals woke up. Immediately after waking, the animals were transferred to the housing scantainer for 1 week of recovery before recording.

**TABLE 1:** Stereotaxic coordinates of the implanted electrodes.

Regions	Coordinates (A/L/V)
S1c	−1.06/−1.4/−0.7
S1i	−1.06/+1.4/−0.7
PACc	−2.06/−1.5/−0.5
PACi	−2.06/−1.5/epid
VPLc	−1.7/−1.8/−3.7
VPLi	−1.7/+1.8/−3.7
ACCc	+1.98/−0.35/−1.7
ACCi	+1.98/+0.35/−1.7
Insc	−1.06/−3.7/−3.5
Insi	−1.06/+3.7/−3.5
AMYGc	−1.06/−2.2/−4.7

*Numbers in mm from bregma, according to Paxinos and Franklin (2001).*

### Stimulation, Electrophysiology, and Behavior

**FIGURE 5:**
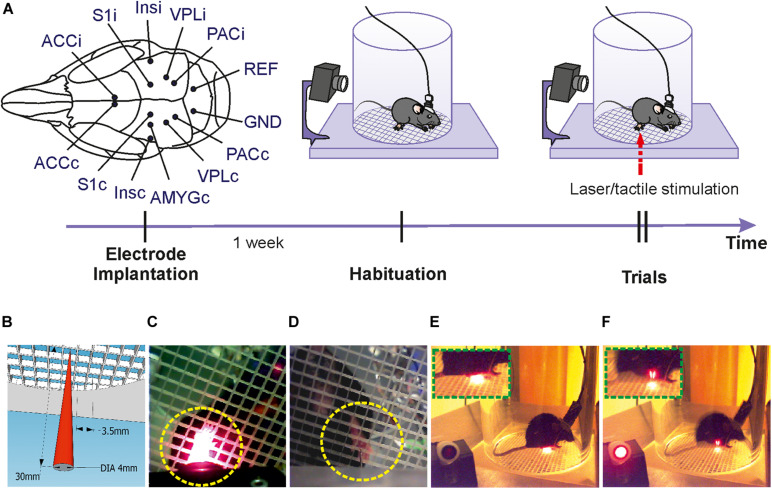
Experimental procedures. **(A)** Upper left: Sites of electrode implantation (scheme showing insertion points for all electrodes used in all mice). Upper right panes and time arrow: time course of experiments. One week following electrode implantation several habituation recordings were done before recording responses to laser/tactile stimulations. **(B)** Schematic illustration of the stimulation laser with initial diameter of 4 mm and about 50 μm at the focal plane (at 30 mm distance between the stimulator and the grid plate). The length of each grid field is 3.5 ms, which is much larger than the focal diameter of the laser beam. This ensures for reliable targeting of the stimulus after positioning the pilot laser manually through the middle of one of the grid holes. **(C)** Targeting of right hind paw by laser light (view from bottom camera). **(D)** Targeting of right hind paw by tactile stimulator. **(E)** View of the lateral camera showing a mouse in resting state. **(F)** View of the lateral camera showing the moment of paw withdrawal following laser stimulation.

One week after surgery, mice were placed in the behavioral platform and the recording cable was connected with the headstage. Habituation to the recording situation was done for 1 h per day for 2–3 days (Figure 5A). A cardboard partition was placed between the apparatus and the experimenter to prevent visual contact. Local field potentials (LFPs) were amplified (RHA2116, Intan Technologies), filtered (1–500 Hz), digitized at a rate of 2.5 kHz, and stored on a computer for off-line analysis.

Experimental trials were conducted after habituation. The indicator LED was turned toward the lateral camera for video recording. Before the real stimulation, both mechanical and laser stimuli were tested without targeting on the mouse (targeting the empty space of the grid plate), to make sure that the stimulation worked and that the red light of the indicator LED did not evoke any paw withdrawal or other pain-like behaviors. With the guidance of the bottom camera, stimulation could be reliably targeted through the grid plate onto the hind paw area of the mouse. Tactile stimulations were applied 3–6 times onto the right hind paw of the mouse while it was awake and immobile. Then, the tactile stimulator was replaced by the laser which was used to stimulate the right hind paw for 3–6 times. In order to apply different energies we applied laser pulse durations of different durations: 3 ms (2.3 mJ), 5 ms (5 mJ), and 10 ms (11.7 mJ). These pulses are far shorter than the subsequent activation of central neuronal networks or behavioral reactions, allowing for appropriate alignment of evoked potentials. Stimulations were done when the animal was immobile such that the paw could be targeted. The interval between two stimuli was dependent on the behavior of the animal. Only when the mouse was awake and resting quiescently on its four paws, the stimulation was applied. In many cases the mouse was actively exploring the chamber, such that intervals between stimuli lasted as long as half an hour or longer. The shortest interval between stimuli was set at 30 s (Ljungquist et al., 2016; Xia et al., 2016). Pain-like behaviors were recorded via the lateral camera from 30 s to 1 min before the stimulation until 30 s after the stimulation. After each experiment, the chamber and the grid plate were cleaned with tap water and ethanol, and the stimulators and the scaffold were cleaned with ethanol to remove any smells that could affect behavior in subsequent recordings.

### Histology

After completing the experiments the animals were anesthetized by intraperitoneal injection of an overdose of pentobarbital (100 mg/kg) followed by transcardial perfusion with phosphate-buffered saline (PBS) followed by 4% paraformaldehyde (PFA, solved in phosphate buffer, Sigma, Germany). The brains were carefully removed and stored in 4% PFA at 4°C for a minimum of 2 days. We then cut coronal slices of 50 μm thickness, using a Vibratom (Leica VT 1200S). Slices were stained with Cresyl Violet (Nissl staining) and inspected by light microscopy to verify the electrode position.

### Data Analysis

The evoked potentials were analyzed with custom-written MATLAB routines (Math Works, United States) after averaging 3 – 6 raw traces of LFPs from each animal. Responses to stimulations showed a typical and reproducible pattern of positive and negative peaks. The first positive peak (P1) was, however, small and variable such that it was not used for systematic analysis. Amplitudes of subsequent peaks (N1, P2, and N2) were measured from baseline. Peak-to-peak amplitude was calculated as P2–N1. Data are presented as mean values and standard error of the mean (SEM).

Pain ratings were performed by evaluating pain-related behaviors in video recordings within 30 s after the stimulation which was marked by the indicator light (Figures 1, 5D,E). For tactile stimulation, there was a 24 ms delay between the trigger and physical contact of the actuator with the hind paw. This offset was subtracted from the recorded data for correct alignment, leading to shorter latency times for tactile as compared to laser stimuli (Figure 9C). Percentage and latency of right hind paw withdrawal were recorded via the lateral camera. The stimulation was only applied when the mice were resting in an immobile position on all four paws. Withdrawal was normally restricted to the stimulated paw (right hind paw). Withdrawal latency measured the time from the beginning of the stimulus to the first detectable withdrawal of the right hind paw. In addition, stimulation-induced guarding, freezing and flinching were summarized as pain-related behaviors (Luger et al., 2002; Deuis et al., 2017). Guarding refers protection of the right hind paw by lifting or reducing weight on it while moving or standing; freezing is defined as suppression of all movements other than those necessary for respiration; flinching describes fast shaking of the hind paw. The occurrence of guarding, freezing and flinching was used as a pain score [modified from Abdus-Saboor et al. (2019)]. For example, animals with two of the three behaviors got a score of two for the particular trial. Behavioral responses of the animals were counted separately for each trial (3 - 6 stimulations) and averaged.

**FIGURE 6:**
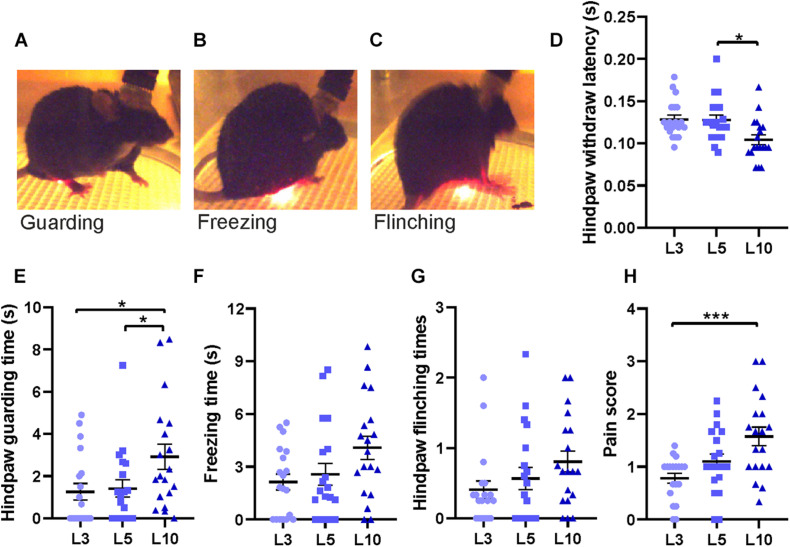
Behavioral responses to laser stimulations of different durations. **(A)** Hind paw withdrawal, **(B)** freezing, **(C)** flinching, and **(D)** latency to hind paw withdrawal for different laser stimulus durations (averaged data from 3 – 6 trials for each data point). **(E)** Guarding time following laser stimulation for 3 ms (L3), 5 ms (L5), and 10 ms (L10). **(F)** Freezing time for different laser stimulations. **(G)** Number of hind paw flinching. **(H)** Pain score combining guarding time, freezing time and flinching times (for details, see section “Methods”). Horizontal bars and whiskers show mean and SEM. Friedman test with Dunn’s multiple comparisons test for **(D,F,G,H)** and **H**; repeated measures of one-way ANOVA with Tukey’s multiple comparisons test for **(E)**, *n* = 19 mice, *p < 0.05, ***p < 0.001.

**FIGURE 7:**
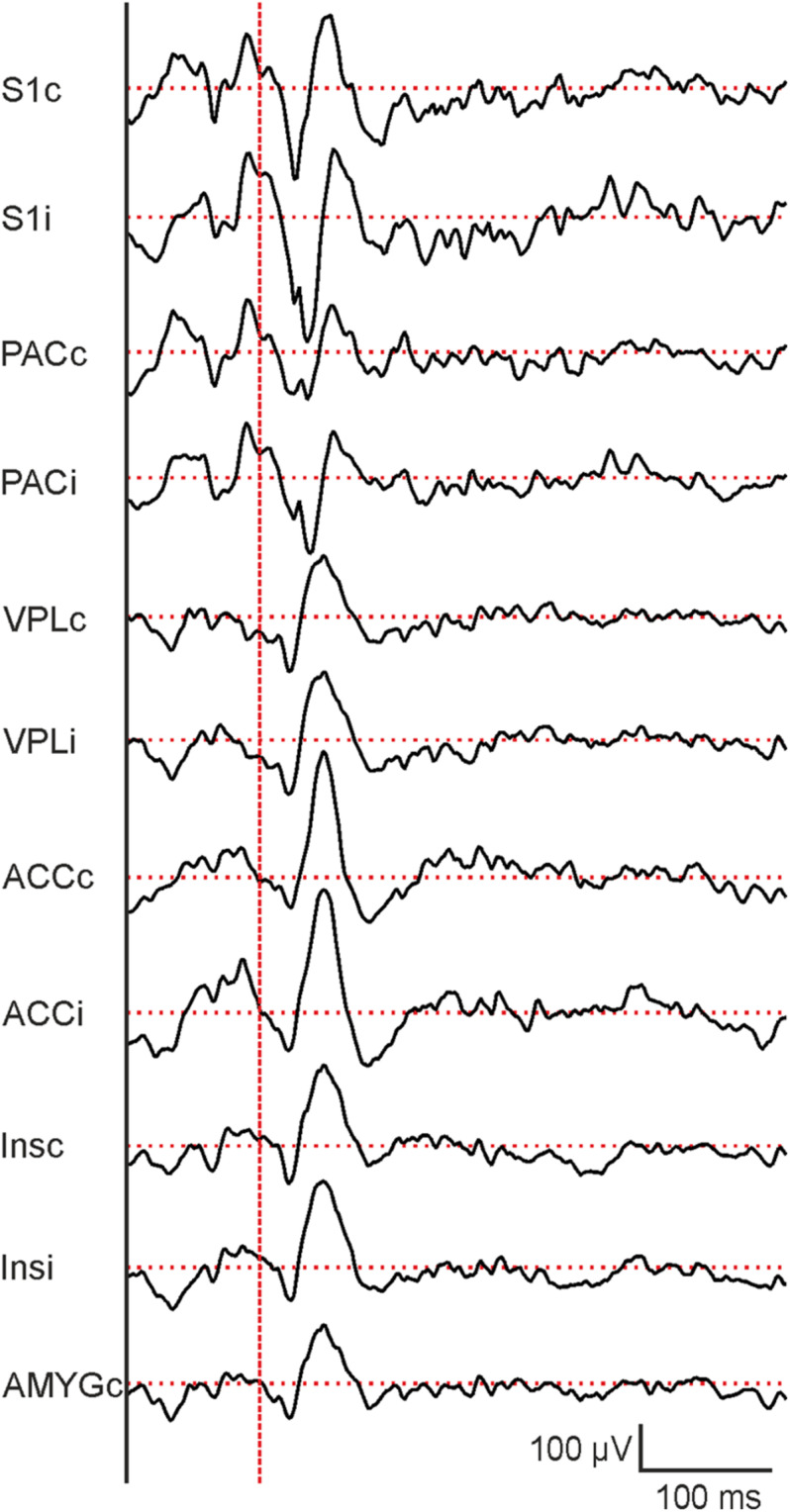
Representative evoked potentials from different brain regions following 10 ms laser stimulation. From top to bottom: contralateral and ipsilateral primary somatosensory cortex (S1c and S1i), parietal cortex (PACc and PACi), ventral posterolateral thalamic nucleus (VPLc and VPLi), anterior cingulate cortex (ACCc and ACCi), ventral posterolateral thalamic nucleus (VPLc and VPLi), posterior insula (Insc and Insi) and central nucleus of the amygdala (AMYGc). Vertical dashed red line indicates the beginning of laser stimulation. Horizontal dashed red lines indicate baseline potential.

**FIGURE 8:**
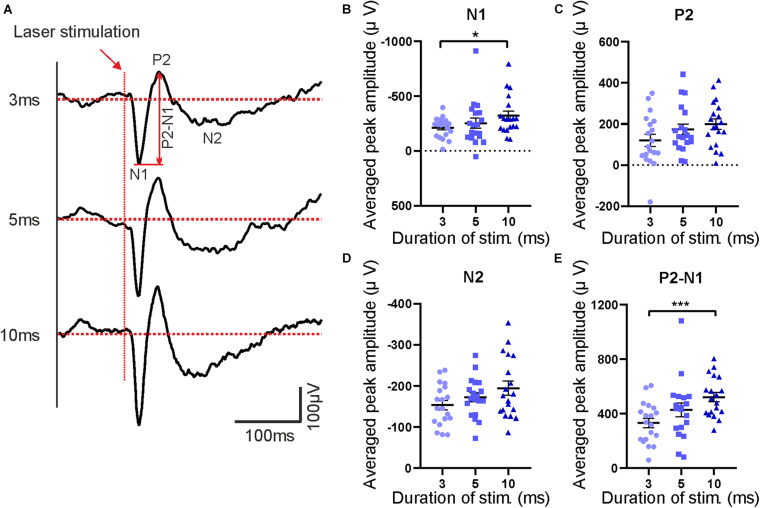
Features of evoked potentials following laser stimulation. **(A)** Averaged laser-evoked potentials in contralateral S1 for different laser durations (3, 5, and 10 ms). Red arrow indicates beginning of the stimulation pulses. The first negative peak (N1), the second positive peak (P2) and the second negative peak (N2) are marked in the upper trace. **(B–E)** Averaged peak amplitudes of N1, P2, N2, and P2–N1, respectively. Bars show mean with SEM. Friedman test with Dunn’s multiple comparisons test, *n* = 19 mice, *p < 0.05, ***p < 0.001.

**FIGURE 9:**
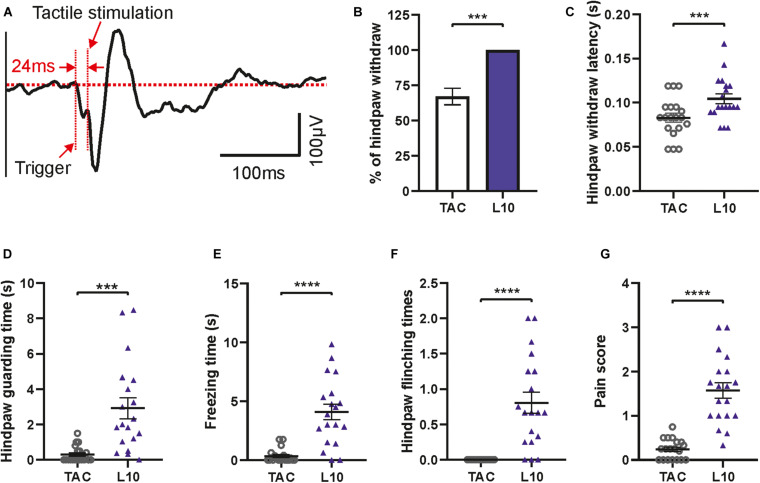
Evoked potential and behavioral features following tactile stimulation. **(A)** Averaged tactile stimulation-evoked potential in contralateral S1. Note the delay between trigger and response. Diagrams show significant differences between tactile stimulation (TAC) and 10 ms laser stimulations of hind paw (L10). **(B)** Percentage of paw withdrawal reactions. **(C)** Hind paw withdrawal latency. **(D)** Guarding time. **(E)** Freezing time. **(F)** Flinching times. **(G)** Pain score for tactile stimulation versus 10 ms laser stimulation. Bars show mean with SEM. Wilcoxon matched-pairs signed rank test for **(B)**, **(C)**, **(E)**, **(F)**, and **(G)**; two-tailed paired *t*-test for *D*, *n* = 19, ***p < 0.001, ****p < 0.0001.

### Statistics

Data are presented as mean values ± SEM. Data normality was tested by Anderson-Darling test. For normally distributed data, repeated measurement of one-way ANOVA was used with Tukey’s multiple comparisons test for comparisons of more than three groups, and two-tailed paired *t*-test for comparisons of two groups. Non-parametric tests were used for non-normal data. Friedman test with Dunn’s multiple comparisons test was used for comparison of more than three groups, while Wilcoxon matched-pairs signed rank test was used for comparison of two groups. Significance of differences is indicated with asterisks (^∗^*p* < 0.05, ^∗∗^*p* < 0.01, ^∗∗∗^*p* < 0.001, ^****^p < 0.0001).

## Results

We developed an apparatus for measuring precisely timed electrophysiological and behavioral responses to laser-induced and tactile stimulation in freely moving mice (Figures 1A,B). The animal was located in a custom-made chamber on top of a grid, giving access to the paw from underneath (Figures 1A, 2A,B). The apparatus was tested for painful heat stimuli, using a laser beam, and for tactile stimulation with an electrically driven mechanical actuator (Figure 2E). The position of the laser beam and the actuator can be manually controlled by a control stick under guidance by a video-image from underneath the animal (Figures 1A, 2C). Evoked potentials were recorded from previously implanted electrodes in brain regions of interest. Electrophysiological recordings and the stimulation signals were synchronized by a trigger signal from a custom-made control unit (Figure 2D). Behavior was monitored with a lateral HD-camera fixed besides the chamber (Figure 1A). Within the device, different stimulators can be flexibly mounted as long as they have similar outer dimensions. Here, a laser and a mechanical stimulator have been used as shown in Figure1B.

We tested the setup in a series of experiments with mice that had been previously implanted with electrodes in different brain regions. Recordings of LFPs and behavioral responses toward stimuli began after recovery from surgery and habituation to the experimental chamber (Figure 5A). The laser beam was guided with help of a bottom camera allowing to target the right hind paw without restricting movements of the mouse (Figures 5B,C). A lateral camera recorded the animal’s behavior before, during and until 30 s after stimulation (Figures 5D,E). The indicator LED beside the chamber shined red light toward the lateral camera when stimulation was triggered, to facilitate the alignment of the stimulation and the behaviors via video. Preliminary tests (targeting the laser onto the empty space of the grid plates) showed that the indicator LED did not evoke any paw withdrawal or pain-like behaviors when stimulation was not targeted on the animal.

To test the function of the custom-made apparatus, laser stimuli of different durations (3 ms, 5 ms and 10 ms) were applied. This corresponds to different energy transfer into the targeted tissue, ranging from 2.3 to 11.7 mJ (∼1.5 – 6 mJ/mm^2^; see Mitchell et al., 2014; Rosenberger et al., 2020). Stimuli of all durations were painful when directed to a human finger, and all pulses led to hind paw withdrawal in every single trial (data not shown). Latency of hind paw withdrawal decreased significantly from 5 to 10 ms illumination time, indicating that intensity of painful stimuli can be varied by stimulus duration (Figure 6D). Following paw withdrawal, mice frequently returned their hind leg to the grid in a guarding manner, holding the paw aloft, or reduced ground contact to the heel or toes of the paw while resting or moving (Figure 6A). We also observed typical freezing behavior with no visible movements except for respiration (Figure 6B). In some trials, mice showed several fast paw shakes called “flinching” (Figure 6C). These behaviors can serve as a behavioral measure of pain sensitivity. Time of guarding and freezing, as well as times of flinching within 30 s after different laser stimulation durations were measured based on video recordings, respectively (Figures 6E–G).

There was a significant increase of hind paw guarding time after 10 ms laser stimulation compared to 3 or 5 ms (Figure 6E). Freezing and flinching, although showing a graded response to different durations of laser stimulation, did not reach statistically significant differences (Figures 6F,G). Behaviors were very variable between trials such that we quantified the integrative effect of pain sensitivity by a simplified pain score, counting the occurrence of guarding, freezing or flinching of each animal in each trial (values 0 – 3). This score was significantly larger at 10 ms laser stimulation compared to 3 ms, indicating increased pain sensation (Figure 6H). Therefore, the apparatus is able to induce and record graded heat-induced pain effects based on simple behavioral measures.

In parallel to behavioral recordings, laser-evoked LFPs were recorded from different brain regions, including the contralateral somatosensory cortex (S1) which serves as an example in subsequent descriptions. Sample raw traces of evoked potentials are shown in Figure 7. Responses to individual trials at each laser duration (3, 5, and 10 ms, respectively) were averaged before analysis (Figure 8A) and quantified as amplitudes of the first negative peak (N1), the second positive peak (P2), and the second negative peak (N2) as well as peak-to-peak amplitude (P2-N1; Figure 8). Similar to behavioral responses, a significant increase of N1 and P2-N1 amplitude was observed for increased laser stimulation times between 3 and 10 ms (Figures 8B,E). P2- and N2-amplitudes did not change with increasing laser duration (Figures 8C,D). Peak latencies of N1, P2, and N2 did not change with laser pulse duration (data not shown). Together, electrophysiological and behavioral measurements yielded consistent, comparable and graded responses to an acute painful stimulus.

As a control we tested responses to non-painful, tactile stimuli applied by a mechanical actuator with a blunt needle tip (Figures 1B, 2E). This custom-made device had the same dimensions as the laser stimulator, facilitating integration into the apparatus (Figure 2E). A 24 ms delay (due to the mechanical trigger, see section “Methods”) was subtracted from the latencies of evoked potentials (Figure 9A) and of paw withdrawal (Figure 9C) for correct alignment. Evoked potentials following tactile stimulation were measured and averaged in the same way as described for laser stimulation (Figure 9A). We found reliable N1-, P2-, and N2-responses in S1 with a mean delay of 24 ms between the trigger time and touch of the mouse hind paw. Hind paw withdrawal occurred only in 68% of trials which is significantly lower than the reaction to laser stimulation (100%, i.e., withdrawal upon each stimulus; Figure 9B). Compared to 10 ms laser stimulation, the hind paw withdrawal latency after 24 ms corrections was significantly shorter by tactile stimulation (Figure 9C), indicating faster transmission of tactile signals than pain. In addition, tactile stimulation showed significantly lower values in other pain-like behavioral response measures: guarding time (Figure 9D), freezing time (Figure 9E) and flinching times (Figure 9F). All these values indicated a reduced salience of stimulus, except the shorter withdrawal latency which was, however, determined by the different delay and nature of the stimulus (see discussion). Moreover, the integrative pain score of tactile stimulation was also much lower than that of laser stimulation (Figure 9G). Thus, the custom-made apparatus can be used to measure responses to mechanical stimuli as well as to painful stimulations.

## Discussion

We describe an apparatus for laser and tactile stimulations in freelybehaving mice, suited for simultaneous recording of evoked potentials and behavioral responses. The device allows for manual or automatic triggering of laser- or other kinds of stimulations (for example tactile), leading to precisely timed and standardized electrophysiological and motor responses (Figures 1, 2, 5). The trigger signal of the stimulus is synchronized with LFP recordings by a custom made control unit, while behavioral reactions are recorded by a lateral camera which is synchronized with the stimulus by an indicator-LED light. Behavioral responses (Figure 6) and evoked potentials (Figures 7, 8) show significant differences between short (3 ms) and long (10 ms) laser stimulations, showing that stimuli of different pain intensity can be applied. Furthermore, the apparatus is able to carry other types of stimulators, as demonstrated with the tactile stimulation by a custom built stimulator in our experiments (Figure 9). In this case, behavioral parameters show a significantly smaller reaction as compared to laser stimulation, indicating a good differentiation and reproducible measurement of different kinds (pain/non-pain and heat/mechanical) of stimulation by the apparatus.

Evoked potentials have long been studied and somatosensory evoked potentials have been routinely used as reliable test in both human research and clinical diagnostic practice (Chiappa and Ropper, 1982). Laser evoked potentials were broadly adopted for thermal pain studies in humans (Treede et al., 2003). For example, brief painful infrared lasers were established and validated as a nociceptive stimulus in humans (Bromm and Treede, 1984; Plaghki and Mouraux, 2003). Laser evoked potentials can be easily recorded by human EEG or ECoG for pain study (Bromm and Treede, 1991; Cruccu and Garcia-Larrea, 2004; Ohara et al., 2004). Nonetheless, human studies have strong limitations in experimental pain research, for example the lack of recordings from subcortical brain regions. For this and further practical and ethical reasons, animal experiments are indispensable in the field. Laser-evoked potentials are frequently used for pain induction, as they are non-invasive, well reproducible and allow for graded responses by varying stimulus strength or duration (Shaw et al., 1999; Tsai et al., 2004; Albert et al., 2012; Mitchell et al., 2014; Peng et al., 2018). These cues can, in principle, be applied to awake and unrestraint animals, allowing for parallel assessment of behavioral and electrophysiological responses. Such studies are, however, not frequently performed in mice due to the practical difficulties in targeting laser stimuli (Chaplan et al., 1994). While imposing restrictions of movement on the animal can facilitate the stimulation, it may cause additional stress and affect both neuronal and behavioral responses, especially when the stimulation is noxious. Our custom built device is able to leave the mouse relatively unrestrained within a small chamber (120 mm diameter), allowing for free movements and reliable stimulation of the hind paw at the same time. Stress is further reduced by the cardboard partition placed between the animal and the experimenter, preventing visual contact. In principle, the device can even be operated without physical presence of the experimenter, using automatic triggering of stimuli. This is, however, hampered by the difficulty to position the device and would require either a complex two-dimensional positioning control or mechanical restraint of the animal. In our hands positioning of the stimulator by the experimenter proved as a reliable and practical solution.

Evoked potentials constitute a network-level correlate of neuronal activity following the respective cue, in our case a painful laser (heat) stimulus. They do, however, not allow to unambiguously define the underlying mechanisms. Painful stimuli are transmitted to the central nervous system via Aδ- and C-fibers, and they induce activity in multiple cortical and subcortical areas (Apkarian et al., 2005; Gross et al., 2007; Dowman et al., 2008; Zhang et al., 2012). In peripheral sensory nerves, infrared laser stimulation can simultaneously activate Aδ- and C-fiber receptors (Bromm and Treede, 1984; Truini et al., 2004; Mitchell et al., 2014). As a general rule, Aδ fibers respond particularly to rapidly changing temperatures (as applied in our study) while C-fibers are optimally activated by slow rates of heating (Yeomans and Proudfit, 1996; Hsieh et al., 2015). This difference in response dynamics is consistent with the faster conduction velocity of Aδ-fibers (3 – 30 m/s) as compared to C fibers (0.5 – 2 m/s). With these velocities, a rough estimate for 10 cm conduction distance would yield delays of 0.003∼0.03 s for Aδ-fibers versus 0.05 – 0.2 s for C-fibers. The prominent N1-peak in our evoked potentials occurred at ∼ 0.02 s, consistent with values reported for Aδ–mediated responses in previous work (Xu et al., 2015; Uhelski et al., 2017).

In addition to evoked neuronal network responses, our custom built device is also capable to record behavioral features. There are several animal models for heat stimulus-evoked pain tests, such as the hot plate test, the Hargreaves test, the thermal probe test and others (Deuis et al., 2017). The hot plate test is designed to measure the overall pain-induced movement (Eddy and Leimbach, 1953), while the Hargreaves test does allow more specific behavioral measurements in unrestrained animals, esp. withdrawal of the hind paw of mice or rats (Hargreaves et al., 1988). However, the Hargreaves test is normally only used for pure behavioral experiments without simultaneous electrophysiological recordings. Combining both signals is difficult, since the paw withdrawal latency in Hargreaves tests is relatively long (seconds), preventing exact alignment with evoked potentials which have a time resolution of milliseconds. Our custom built apparatus solves this problem by using highly intense but short laser pulses of few milliseconds (3, 5, and 10 ms). These stimulations are strong enough to evoke pain but short enough to synchronize the resulting evoked potentials. Intense short laser pulses are also used in human studies, facilitating comparison (Bromm and Treede, 1984; Greffrath et al., 2007). In addition to paw withdrawal, we also measured other pain-induced behaviors like guarding, flinching and freezing (Luger et al., 2002; Tappe-Theodor and Kuner, 2014; Pitzer et al., 2016). This requires real-time video recording of the stimulation process (Abdus-Saboor et al., 2019) which was achived by a HD-camera mounted laterally besides the chamber. Time of stimulation was indicated by a LED, allowing accurately measurement of paw withdraw latency and further behavioral responses.

Our device can easily be used for other modalities, for instance tactile stimulation. Here, the von Frey Hair test is a commonly used measure of increased sensitivity like hyperalgesia or allodynia (Pitzer et al., 2016). However, the classical von Frey hair test largely relies on the skills of the experimenter in targeting and gradually increasing force on the mouse paw (Yoburn et al., 1984; Minett et al., 2012). Furthermore, similar to Hargreaves’ test, time to paw withdrawal is relatively long (seconds), hampering accurate alignment with evoked potentials. In the present study, we used a custom made tactile stimulator with a blunt needle tip for non-painful stimulation. Intensity can be controlled by a power supply unit (PSU) which, at sufficient voltage, results in a reproducible delay between triggering of the device and physical contact to the paw. The actuator has the same size as the laser stimulator to facilitate exchange between both devices during the experiment. For the same reason, both share the same control unit for triggering. Our results reveal reliable measurements of tactile responses both as evoked potentials and as behavioral reactions. The low pain score compared to laser stimulation shows that the apparatus can generate painful as well as non-painful (control) stimuli.

Despite of the above mentioned advantages, our apparatus has some limitations. In humans, evoked potentials display habituation if the stimulation is restricted to the same location (Greffrath et al., 2007). However, in freely moving mice, it is almost impossible to hit precisely the same location of the hind paw in each trial. This may increase variance of results due to unreliable occurrence or absence of habituation, depending on the precise target region on the hind paw. We tried to reduce this effect by limiting the number of trials per animal to 3–6, and by using inter-stimulus intervals of at least 30 s (Ljungquist et al., 2016; Xia et al., 2016). In most cases, intervals were much longer (see section “Methods”). Secondly, it is not yet possible to define an evoked potential which specifically indicates pain (Mouraux and Iannetti, 2008, 2009; Hu and Iannetti, 2016). In our study, evoked potentials to tactile or laser stimulation, respectively, were similar, such that any distinction between painful and non-painful sensations does still rely on behavioral readouts. In addition, it is likely that the evoked potentials reflect electrographic activity of both, sensory and motor networks, including sensory-motor feedback loops. Evoked potentials should therefore be interpreted with caution and possibly be addressed as sensory-motor responses. This holds true even in the absence of visible major movements. Untangling sensory and motor components is only possible to a limited degree, esp. in higher-order associational networks of the pain matrix (Apkarian et al., 2005; Gross et al., 2007; Dowman et al., 2008; Zhang et al., 2012). Together, it remains a major challenge in pain research to define an electrophysiological signature of pain which should, in principle, be possible based on the activation of the pain network (Melzack, 1999; Mouraux et al., 2011; Wager et al., 2016; Ponsel et al., 2020).

In conclusion, we present here an apparatus which combines evoked potential recordings as well as behavioral measurements in freely moving mice. Heat (laser) pain stimulation and mechanical tactile stimulation can be applied and differentiated. The apparatus can be applied to a variety of experimental paradigms, including chronic pain, or pain sensitivity in different disease models in mice. The custom built device can help to study functional mechanisms of pain and compare them with non-painful somatosensory responses in freely moving mice.

## Data Availability Statement

The original contributions presented in the study are included in the article/supplementary material, further inquiries can be directed to the corresponding author/s.

## Ethics Statement

The animal study was reviewed and approved by the Governmental Supervisory Panel on Animal Experiments of Baden Württemberg, Karlsruhe (35-9185.81/G-115/14).

## Author Contributions

AD and JB conceived and designed the experiments. YY supervised all experimental work involving animals. JZ performed the experiments. YY and JB supported and analyzed the data. All electronic components of the apparatus were designed, built, and tested by LE. All authors participated in wrote the manuscript which was originally drafted by JZ and AD.

## Conflict of Interest

The authors declare that the research was conducted in the absence of any commercial or financial relationships that could be construed as a potential conflict of interest.
